# Pediatric age estimation from radiographs of the knee using deep learning

**DOI:** 10.1007/s00330-022-08582-0

**Published:** 2022-03-01

**Authors:** Aydin Demircioğlu, Anton S. Quinsten, Michael Forsting, Lale Umutlu, Kai Nassenstein

**Affiliations:** Department of Diagnostic and Interventional Radiology and Neuroradiology, University Hospital Essen, University of Duisburg-Essen, Hufelandstr. 55, D-45147 Essen, Germany

**Keywords:** Knee joint, Bone age measurement, Radiography, Pediatrics, Deep learning

## Abstract

**Objectives:**

Age estimation, especially in pediatric patients, is regularly used in different contexts ranging from forensic over medicolegal to clinical applications. A deep neural network has been developed to automatically estimate chronological age from knee radiographs in pediatric patients.

**Methods:**

In this retrospective study, 3816 radiographs of the knee from pediatric patients from a German population (acquired between January 2008 and December 2018) were collected to train a neural network. The network was trained to predict chronological age from the knee radiographs and was evaluated on an independent validation cohort of 423 radiographs (acquired between January 2019 and December 2020) and on an external validation cohort of 197 radiographs.

**Results:**

The model showed a mean absolute error of 0.86 ± 0.72 years and 0.9 ± 0.71 years on the internal and external validation cohorts, respectively. Separating age classes (< 14 years from ≥ 14 years and < 18 years from ≥ 18 years) showed AUCs between 0.94 and 0.98.

**Conclusions:**

The chronological age of pediatric patients can be estimated with good accuracy from radiographs of the knee using a deep neural network.

**Key Points:**

• *Radiographs of the knee can be used for age estimations in pediatric patients using a standard deep neural network.*

• *The network showed a mean absolute error of 0.86 ± 0.72 years in an internal validation cohort and of 0.9 ± 0.71 years in an external validation cohort.*

• *The network can be used to separate the age classes < 14 years from* ≥ *14 years with an AUC of 0.97 and < 18 years from* ≥ *18 years with an AUC of 0.94.*

**Supplementary Information:**

The online version contains supplementary material available at 10.1007/s00330-022-08582-0.

## Introduction

Age estimation by radiological methods is performed by assessing the skeletal maturity in scans and has applications in many different contexts: In forensic medicine, the aim is to identify the age of unknown deceased persons [1], whereas in legal applications, the goal is to determine whether an adolescents with dubious date of birth is of legal age [2]. In pediatric endocrinology, bone age estimation is commonly used to determine whether a growth disorder is present or not [3].

Various radiological approaches have been proposed for bone age estimation ranging from radiographs of the hand [4], elbow [5, 6], knee [7], pelvis [8], or feet [9], computed tomography (CT) of the teeth [10], clavicle [11], or rib [12] to magnetic resonance imaging (MRI) of the knee [13], hand [14], or iliac crest [15]. Although undoubtedly bone age estimation from radiographs of the left hand is by far the most commonly used in clinical routine, this method has some limitations: The most frequent used reference for this is the Greulich and Pyle (G&P) atlas, which is based on single radiographs of the left hand taken more than 100 years ago from a population in Cleveland, OH, consisting primarily of white children of high socioeconomic status, raising the question how well these data can be transferred to current populations.

Another method for bone age estimation is based on radiographs of the knee. The knee appears well suited for age estimation for several reasons: First, the knee yields information for three epiphyses (the distal femur, proximal tibia, and proximal fibula); second, the knee is easy to x-ray in a well-defined position with low radiation exposure; and last but not least, large current case series of knee radiographs can easily be created, as the knee is frequently examined in daily clinical practice in the context of trauma. Pyle and Hoerr created a reference atlas for bone age estimation from knee radiographs that can be similarly used as the well-known Greulich-Pyle atlas [7]. The atlas was verified in certain populations and has been shown to be rather precise, although small deviations could be seen [16–19]. O’Connor proposed a more systematic way to determine the age by introducing ten maturity indicators and subsequent regression over the estimated ages for each indicator [20].

Since the age estimation, based either on an atlas or on maturity indicators, is time consuming and also prone to intra- and inter-rater variability [21], an automation would be of interest, because this would reduce the time effort while at the same time lead to more standardization. Accordingly, deep learning methods [22] based on artificial neural networks have been employed for automation of age estimation based on radiographs of the hand [23]. A similar automation for age estimation based on radiographs of the knee is currently missing. Therefore, in this study, we apply deep learning methods to demonstrate that such automation is possible in a German population.

## Materials and methods

Ethical approval for this retrospective study was granted by the local ethics committee (Institutional Review Board of the University Hospital Essen; registry number 21-10069-BO). Written and informed consent was waived by the ethics board because of the retrospective nature. All methods and procedures were performed in accordance with the relevant guidelines and regulations.

### Patients

Using the radiological information system of our hospital (University Hospital, Essen, Germany), all patients younger than 21 years who had a radiograph of the knee in anterior-posterior direction between January 2008 and December 2020 were collected. Two cohorts were created, patients with an examination between January 2008 and December 2018 formed the training cohort, while those between January 2019 and December 2020 were used as the validation cohort. Since patients present in the training as well as in the validation cohort can introduce positive bias, scans of patients included in the training cohort were removed from the validation cohort so that each patient was included in exactly one of the two cohorts. Scans were excluded if they were mislabeled and showed a lateral view. They were also excluded if they did not show the full knee between the distal femoral physis and the proximal fibular physis (e.g., a scan was excluded if the femur was of main interest of acquisition and the fibula was thus not visible), or if a knee arthroplasty had been performed. Scans with screws or temporary stabilization artifacts were not excluded, as long as they did not occlude a major part of the knee. Furthermore, if the image quality was deemed too low, e.g., if the scan was under- or overexposed or a cast obstructed the scan too much, the scan was excluded. In case both knees were visible, only one of them was taken at random.

**Fig. 1:**
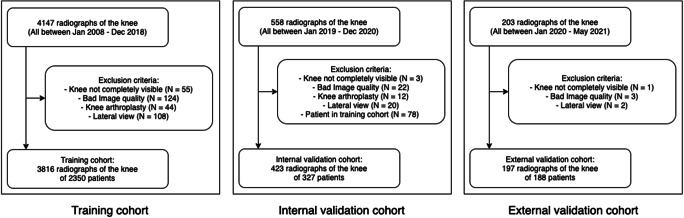
Patient flowcharts with inclusion and exclusion criteria

Based on these criteria, the training set comprised 3816 radiographs from 2350 patients, while the internal validation cohort consisted of 423 radiographs of 327 patients respectively (Fig. 1).

In addition, an external validation cohort was acquired (Elisabeth Hospital, Essen, Germany). Patients with age < 21 years with a radiograph of the knee in anterior-posterior direction between January 2020 and May 2021 were included into the external validation cohort with the same criteria. After applying the exclusion criteria, the external validation cohort comprised 197 radiographs of 188 patients (Fig. 1).

### Radiograph acquisition

All radiographs were acquired mainly on Siemens (Siemens Healthineers), AGFA (AGFA Healthcare), and Canon (Canon Medical Systems) scanners (Table 1). On average, the radiographs were acquired with 65.7 kVp (range: 49.9–76.8), 64.8 kVp (range: 51.8–74.8), and 5.1 mAs (range: 1–42) and 5.2 mAs (range 1–32) in the training and internal validation cohorts respectively. For the external validation cohort these parameters were not available in the DICOM tags.

**Table 1:** Overview of the scanners used for the acquisition of the radiographs. Scanners with less than 50 examinations were gathered into the “Other” group

	All (*N* = 4436)	Train (*N* = 3816)	Internal validation (*N* = 423)	External validation (*N* = 197)
SIEMENS Flurospot Compact FD	2287	1884	403	0
AGFA (CR 58, Solo, 51xx, Compact Plus)	1902	1902	0	0
CANON	189	0	0	189
Other	58	30	20	8

### Collected variables

For each radiograph, the chronological age was computed by taking the difference between the birth date and the acquisition date. Moreover, the sex of the patient was extracted from the DICOM tags and used as additional input to the neural network. For all patients, the birth date as well as the sex information was available.

### Cropping of the knee region

Since the radiographs exhibit a large variety and often only part of the scan actually shows the knee, for efficiency purposes, the images should be centered and cropped roughly the center of ossification, i.e., largely around the intercondylar area. Though manually annotating the area is possible, for a fully automated solution, the area should be cropped also automatically. Accordingly, a network was trained to locate this area by randomly selecting and annotating 1000 knee radiographs from the training cohort by bounding boxes that enclosed the lower part of the femur, the upper part of the tibia, and the upper part of the fibula. The aspect ratio of the box was fixed to 2:3 since the knee is taller than wider. A cascade-CNN was then trained to locate the area. Details of the network and the training procedure can be found in Annex 1. After training, the network was used as a preprocessing tool to crop images around the intercondylar area for all radiographs.

### Preprocessing

**Fig. 2:**
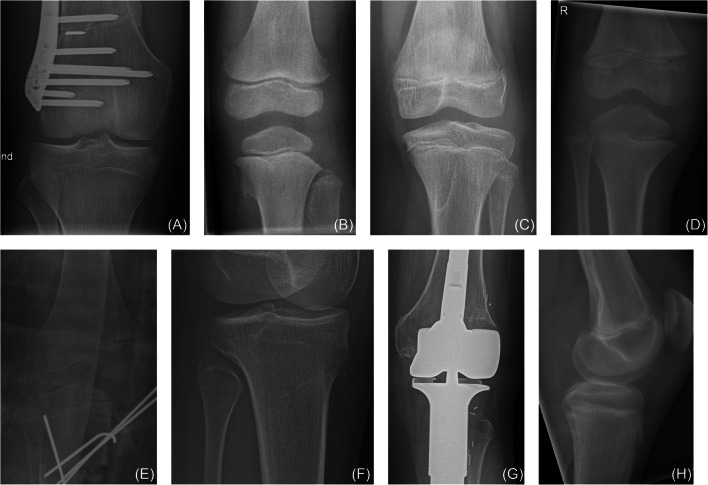
Cropped knee radiographs for three patients. The upper row depicts radiographs that were included into the study while the lower row shows examples of radiographs that were excluded. **A** Male patient (18.5 years). **B** Female patient (3.3 years). **C** Female patient (13.0 years). **D** Male patient (6.8 years). **E** Female patient (15.7 years), excluded because of low image quality. **F** Female patient (5.3 years) excluded because the knee is not fully visible. **G** Female patient (19.8 years) excluded because of knee arthroplasty. **H** Male patient (16.5 years) excluded because of lateral view

The intensity of the cropped images was then linearly rescaled to the range 0–255 (Fig. 2). Images were converted from grayscale to RGB by replicating the gray channel.

### Neural network

A standard network architecture, the ResNet-34, was used for modeling [24]. Because it is well known that sex has a large impact on the maturity of bones, sex was added as a feature to the network. The ResNet-34 was pretrained on the ImageNet dataset [25] and optimized using the L1 loss and the Adam optimizer. During training, several augmentations were used, which regularizes the network and helps its ability to generalize. The batch size was set to 32. Early stopping was employed to avoid overfitting. Details on the network can be found in Annex 2.

### Cross-validation

A 5-fold cross-validation was used during training to optimize the learning rate and to obtain an estimate on the generalizability and performance of the network. The learning rate that obtained the lowest average mean absolute error (MAE) over the cross-validation folds was finally used. Two modeling strategies were tested during cross-validation: using the single best performing model or creating a snapshot ensemble [26]. Snapshot ensembling is a simple technique that boils down to saving the top *k* best performing models that were seen during training and creating a simple ensemble of these by taking the median of their predictions. For this purpose, the best 5 models were saved during training.

### Evaluation

The final model was created by re-using the models trained during cross-validation. The reason for this approach is that it is not as wasteful as an explicit test cohort, which would have to be split off from the training set and would not be used directly in the final modeling. Such a test set would be necessary in our case since the training of the networks used early stopping, which depends explicitly on such a set. In addition, using this approach, every radiograph obtains multiple predictions which could be used as a confidence measure.

The modeling strategy (best model vs. snapshot) together with the best learning rate which showed better results during the cross-validation was selected as the final model.

In addition, the models were evaluated using receiver operating characteristic (ROC) analysis for their ability to distinguish between the age groups < 14 and ≥ 14 years as well as < 18 and ≥ 18 years, which is relevant for forensic applications.

### Statistics

All descriptive statistics were reported as mean ± standard deviation. To compare the absolute mean differences between the true and the predicted ages, a one-sided *t*-test was employed. The null hypothesis for this test is that the absolute differences (corresponding to prediction errors) on average are larger than 1 year; the alternative hypothesis is that the errors on average are smaller than or equal to 1 year. ROC analysis was employed to evaluate separation of age groups. Statistical significance was chosen to be below a *p*-value of 0.05. Correlation coefficients were computed using Pearson’s method. All analyses were conducted with Python 3.7 and the SciPy package.

## Results

### Demographics

**Fig. 3:**
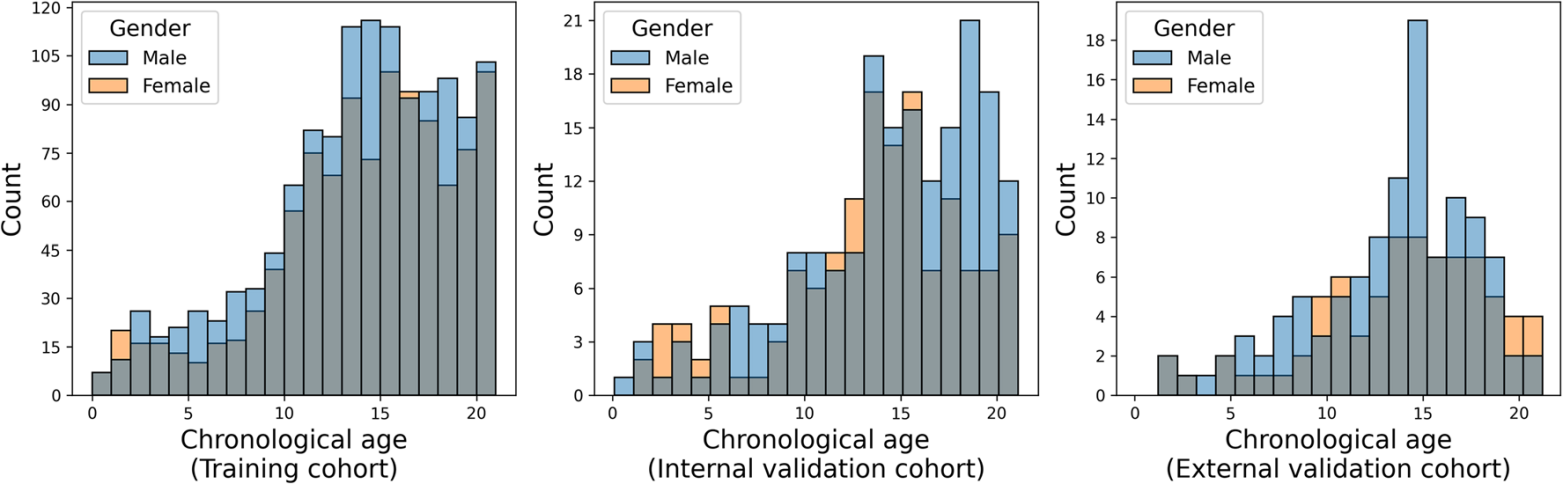
Histogram of the chronological age of all patients. Left: Patients in the training set (*N* = 2350). Middle: Patients in the internal validation set (*N* = 327). Right: Patients in the external validation set (*N* = 188)

The mean age of all patients was 14.0 ± 4.8 years (range: 0–21 years), with 1287 females and 1578 males (Table 2 and Annex 3). No large deviation was seen between the distributions of age and sex between the datasets (Fig. 3).

**Table 2:** Demographics of the patient collective. The *p*-value denotes the significance of a chi-square and a *t*-test for sex and age between the training and the internal and external validation cohorts, respectively

	All	Training cohort	Internal validation cohort	External validation cohort
Gender [F]	45% (1287/2865)	45% (1065/2350)	44% (143/327) (*p* = 0.63)	42% (79/188) (*p* = 0.42)
Age	14.0 ± 4.8 (range: 0–21)	14.0 ± 4.8 (range: 0–21)	14.0 ± 4.8 (range: 0–21) (*p* = 0.92)	13.6 ± 4.4 (range: 1–21) (*p* = 0.12)

### Cropping the knee region

The cropping of the knee region worked with high accuracy; all knees were detected with the exception of 3 radiographs, 2 from younger children (< 2 years), yielding an accuracy above 99%. The errors on the 2 younger patients were not surprising, since there were quite few younger children in the training set, and no attempts had been made to deal with the imbalance.

### Cross-validation

During the cross-validation the best learning rate as well as modeling strategy (best single model vs. snapshot ensembling) was tested. The best learning rate was 10^−4^ together with snapshot ensembling and yielded a MAE of 0.92 ± 0.76 years, although a large difference to the learning rate 9*10^−5^ could not be seen (Table 3). Also, snapshot ensembling showed slightly better results, but again the improvement (0.03 years) against the best single best model was only moderate.

**Table 3:** Mean absolute error (in years) and standard deviation of the models trained during cross-validation. The best absolute value for each modeling strategy is marked in bold

Modeling strategy	Learning rate					
	9*10^−4^	6*10^−4^	3*10^−4^	1*10^−4^	9*10^−5^	6*10^−5^
Single best model	1.01 ± 0.84	0.98 ± 0.8	0.98 ± 0.8	0.96 ± 0.8	**0.95 ± 0.79**	0.97 ± 0.81
Snapshot ensembling	0.97 ± 0.81	0.94 ± 0.79	0.94 ± 0.78	**0.92 ± 0.76**	0.93 ± 0.78	0.95 ± 0.78

Regarding the prediction of the age groups < 14 and ≥ 14 years, the accuracy of the snapshot ensembling model was 0.92, while the AUC was 0.98, with a sensitivity of 0.92 and a specificity of 0.92. For the age groups < 18 and ≥ 18 years, the accuracy of the model was again 0.92, with an AUC of 0.96. The model showed a sensitivity of 0.91 and a specificity of 0.89. Details on the results can be found in Annex 4.

### Internal validation

**Fig. 4:**
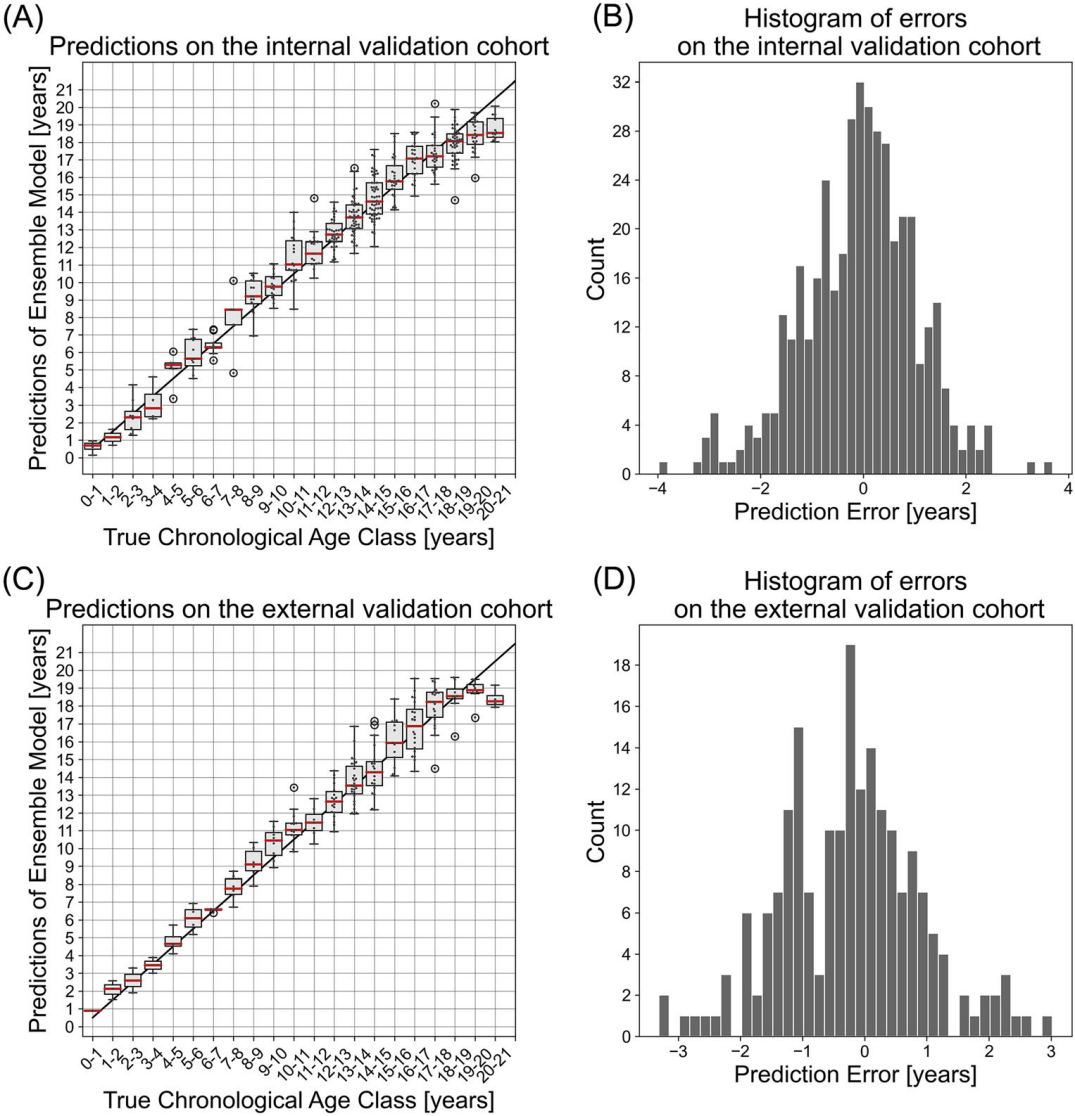
Results of the network evaluated on the validation cohorts. **A** Boxplot for the predictions on the internal validation cohort. **B** A histogram of the prediction errors on the internal validation cohort. **C** Boxplot for the predictions on the internal validation cohort. **D** A histogram of the prediction errors on the internal validation cohort. In the boxplots, for each true chronological age class, a corresponding box with whiskers for the corresponding network predictions was drawn. The median is marked by a red bar, while the whiskers extend to the points inside the 1.5*interquartile range (IQR). In addition, all samples were marked by small dots. Outliers are marked with a circle

Since the snapshot ensemble together with a learning rate of 10^−4^ showed the best results, this combination was used for final modeling. The trained models were then evaluated on the internal validation cohort. The MAE of the model was 0.86 ± 0.72 years (Fig. 4A, B). Compared to the performance during the cross-validation, the MAE was slightly lower. The one-sided *t*-test indicated that the mean of absolute differences between the true and the predicted age is less than 1 year (*p* < 0.001). Accordingly, when comparing the true with the predicted age class, a good correspondence could be seen, although for older patients a gap was visible, where the network underestimates the age for these patients. The correlation coefficient was *R* = 0.97.

**Fig. 5:**
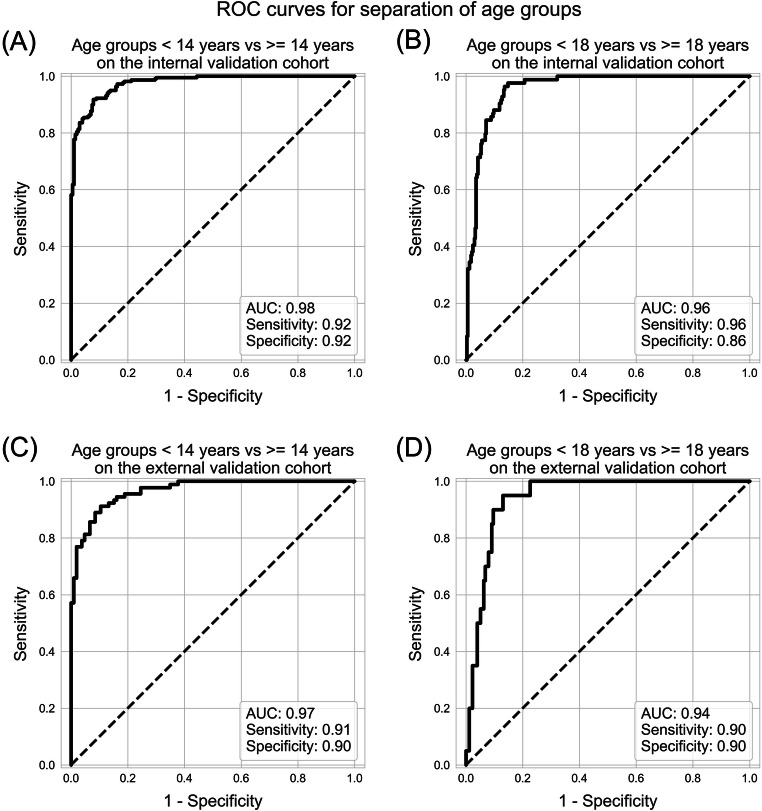
ROC curves for separating the age groups <14 years from ≥ 14 years and <18 years from ≥ 18 years on the validation cohorts. **A** ROC curve for separating the 14-year age groups on the internal validation cohort. **B** ROC curve for separating the 18-year age groups on the internal validation cohort. **C** ROC curve for separating the 14-year age groups on the external validation cohort. **D** ROC curve for separating the 18-year age groups on the external validation cohort

Prediction of the age groups was rather similar to the cross-validation: The model showed an accuracy of 0.90, and an AUC of 0.98 as well as a sensitivity and specificity of 0.92 for separating the 14-year age groups (Fig. 5A, B). Similarly, it showed an accuracy of 0.90, and an AUC of 0.96, a sensitivity of 0.96, and a specificity of 0.86 for the 18-year age groups.

### External validation

When predicting on the external validation cohort, the model yielded a MAE of 0.9 ± 0.71 years (Fig. 4C, D). Similar to the internal validation cohort, the performance was slightly lower than observed during cross-validation. The correspondence between the true and the predicted age class was good as well, and no statistical difference could be seen when testing for a difference smaller than 1.0 years (*p* = 0.017). A tendency to underestimate the age in older patients could be observed as well, similar to the internal validation. Additionally, there seemed to be also a slight overestimation for the age classes between 8 and 11. The correlation coefficient was *R* = 0.97.

Separating the age groups was similar to the validation groups, but showed higher specificity: The accuracy of prediction of the 14-year age groups was 0.90 and the AUC showed a performance of 0.97 with a sensitivity of 0.91 and a specificity of 0.90 (Fig. 5C, D). For prediction of the 18-year age groups, the accuracy was 0.90 as well, with an AUC of 0.94 and a sensitivity as well as specificity of 0.90.

## Discussion

Age estimation in pediatric patients based on the maturity of bones is a commonly used practice for forensic, medicolegal, or clinical purposes. In addition to bone age estimation using radiographs of the hand, which is by far the most commonly used method in clinical routine, bone age can also be estimated from radiographs of the knee. Using the Cleveland study, which started in 1926, Pyle and Hoerr developed an atlas for determining bone age from knee radiographs on a cohort of American children [7]. This atlas has since become a widely used referenced standard and has been subsequently verified in different cohorts [16, 18, 19, 27]. However, more refined estimation methods such as subtle maturity indicators have also been proposed [20].

Because age determination from knee radiographs is quite time consuming and is prone to high inter- and intra-observer variability, automation is of clinical interest. In this study, we have utilized a simple and commonly used deep network to fully automate the chronological age estimation from knee radiographs. The network showed a mean absolute error of 0.86 ± 0.72 years in the internal validation cohort. It was also able to achieve a MAE of 0.9 ± 0.71 years on the external validation cohort, showing that its generalizability could be high. The maximum differences in prediction were 4.0 years and 3.3 years respectively. Correlation of the predictions with chronological age was high, yielding *R* = 0.97 for both validation cohorts. These results are roughly in line with the study of Hackman et al. [17], who used the Pyle and Hoerr atlas in a Scottish population and reported a standard deviation of 0.82 (females) and of 0.90 (males), a maximum difference of 4.3 years, and a correlation coefficient of *R* = 0.95.

Comparing the predicted age classes with the true age classes, the overall fit is rather good, although especially for older patients a larger deviation could be seen. We believe that this stems from the fact that the neural network’s output is normally distributed. Since no patient is older than 21 years, having a mean of around 21 years would yield a higher loss than reducing it to a lower mean. A similar effect should also be present for younger patients, but as both cohorts contained only very few very young patients, the effect is not visible there.

For forensic applications, the age of 14 is essential in some countries since a person becomes legally responsible at that age. Similarly, with the completion of 18 years of age, adult law is authoritative for a person. Because of this, we separated two age groups < 14 and ≥ 14 years and < 18 and ≥ 18 years. Both separations were rather good, showing AUCs of around 0.97 for the 14-year age group and 0.94 for the 18-year group. Despite of this excellent performance, the model does not reach a level high enough for forensic application in a clinical context, where AUCs of at least 0.99 are necessary. The model could be used instead in addition to predictions from other body parts like hands or clavicula to increase overall certainty.

Even though the results are encouraging, radiographs go hand in hand with radiation exposure to the patient and should be avoided especially in pediatric context. A promising alternative to radiographs is MR imaging, since they do not involve any radioactive exposure. Accordingly, in a similar manner as Pyle and Hoerr, Pennock et al. defined an atlas for age estimation in knee MRI [28]. Automation of the age estimation based on knee MRI was proposed by Dallora et al. [13]. They employed a two-step network that first selects the most informative image slice of a given MRI which is then fed to a second network to regress the age based on that slice. The cohort comprised 402 patients of age 14–21, and a 5-fold cross-validation obtained a MAE of 0.793 years for men and 0.988 for women. These MAEs seem to be comparable to the MAEs we have achieved. In a similar study, Auf der Mauer et al. employ a U-Net to segment MRIs into age-relevant anatomical parts, which are then used to regress the age [29]. They report a MAE of 0.69 ± 0.49 using cross-validation on the 175 patients of age 14–21 included into the study as well as an accuracy of 90.6%. Their MAE can be regarded to be better than ours, because if restricted to the same age class 14–21 years, our network achieves a MAE of 0.94 ± 0.74 years and 1.09 ± 0.78 years on the internal and external validation cohorts respectively. Nonetheless, they report that their segmentation of age-relevant parts is of major help as it improves the MAE from 0.97 ± 0.84 years to 0.81 ± 0.65 years (tested only on a single fold of the cross-validation). Thus, our approach might as well benefit from a segmentation of the knee. Another stern difference between our approach and the one by Auf der Mauer et al. lies in the population: They used small, homogeneous study population by including only males with middle to high socioeconomic status with no chronic diseases or severe bone injuries. In contrast, our study population is quite heterogeneous since it comprises all available knee radiographs from the last 12 years. Even though MRI for bone age estimation avoids radiation exposure, it must be noted that MRI has two major disadvantages: the long image acquisition times, which are particularly problematic for very young patients, and the high costs. Therefore, methods based on ultrasound (US) were proposed [30] and a direct comparison between MRI and ultrasound of the knee for age estimation was performed by Herrmann et al. [31]. In their pilot study of only 39 males aged between 14 and 19 years, they showed that MRI and US have a high inter-rater agreement with respect to epiphyseal growth.

As mentioned above, bone age estimation based on radiographs of the left hand is the most common used method in clinical routine. Therefore, automation efforts have been already undertaken for radiographs of the hand, where the current systems are able to produce results as good as those of an expert radiologist, reaching typically error levels of around 4–5 months [32], though recently Gong et al. improved the accuracy substantially and obtained a mean absolute error of less than 2 months [33]. While these results are impressive, a key difference lies in the data used. Hand radiographs often are taken for the task of age estimation, and an extensive amount of work has been put into assembling atlases representing normal growth. It cannot be ruled out that with more effort similar levels of accuracy could be obtained from knee radiographs.

Although the current study has demonstrated that an automated age estimation based on radiographs of the knee is possible, our study shows several limitations. For one, it is well known that aging slightly varies among different populations. While the validations cohorts came from two different but nearby hospitals, they both reflect the same population. Unfortunately, information on ethnicity or socioeconomic status was not available for our study population; therefore, no analysis on the influence of both on the network could be performed.

Although the Pyle and Hoerr atlas uses AP and lateral radiographs for age estimation, we only used radiographs in AP view in our study. The reason for this was to keep the network straightforward. We believe that adding lateral radiographs will improve the estimations. This should be analyzed more extensively in another study with external data from different populations.

Even though machine learning systems and neural networks in particular have shown impressive results, they are still black boxes and their decisions are not readily explainable, making any application in clinical or medicolegal setup arguable, when they are being relied upon without further supervision [34, 35]. A more interpretable approach would be, e.g., to find the important epiphyseal areas in the radiograph and then apply a regression only over these areas. This would make the network decision much more transparent. In addition, we used a non-selected case series, which on the one hand is advantageous, but on the other hand involves the risk that it also includes individual patients who have a growth disorder, which could potentially negatively affect the performance of the network. Last but not least, we used a common network architecture, ResNet-34, which may not be completely optimal for the task at hand, so more research should be done to see if other network architecture may provide even better results.

## Conclusion

The chronological age of pediatric patients can be estimated with high accuracy from knee radiographs using a deep neural network.

## Supplementary Information

ESM 1 (DOCX 20 kb)

ESM 2 (DOCX 51 kb)

ESM 3 (DOCX 706 kb)

ESM 4 (DOCX 691 kb)

## Abbreviations

AUC: Area under the curve
CNN: Convolutional neural network
CT: Computed tomography
MAE: Mean average error
PACS: Picture archiving and communication system
ROC: Receiver operating characteristic

## Declarations

### Guarantor

The scientific guarantor of this publication is Kai Nassenstein.

### Statistics and biometry

Aydin Demircioglu, one of the authors has significant statistical expertise.

### Methodology

• retrospective

• prognostic study

• performed at two institutions
