# Supplementary material for: Pediatric age estimation from radiographs of the knee using deep learning
Source: Eur Radiol. 2022 Mar 1;32(7):4813–22. doi: 10.1007/s00330-022-08582-0 (PMC9213267; doi:10.1007/s00330-022-08582-0)
Supplement: Supplementary file 1 — (DOCX 20 kb) [file 330_2022_8582_MOESM1_ESM.docx]

**Annex 1**

***Annotation network***

For cropping of the area of the knee, a Cascade R-CNN [1] was used. The implementation from the MMDetection framework [2] was employed for this task. As backbone, the ResNeXt-101 was used with cardinality of 64 and bottleneck width of 4d, because this configuration showed better performance (this configuration is called X-101-64x4d-FPN in MMDetection). The network was pretrained on the COCO dataset. All other parameters were left at default, except for the batch size, which was set to 4 because of memory limitations. In more detail, an SGD optimizer with learning rate 0.002, momentum 0.9, a weight decay of 0.0001 was used with a linear warm-up of 500 iterations. At epochs 8 and 11 the learning rate was reduced. As segmentation networks tend to overfit less than other networks, no validation set was employed.

1000 images were randomly selected from the training cohort and converted from DICOM format to 8-bit PNG by simple intensity scaling. Annotation on these images was performed by marking the area of the knee with a bounding box. This box was not chosen to be too tight, because of biological variations, because a too tight box would possibly yield a bounding box that would cut parts of the knee during inference. In addition, the neural network will perform random crops and would already crop the knee during training.

1. Cai Z, Vasconcelos N (2019) Cascade R-CNN: High Quality Object Detection and Instance Segmentation. ArXiv190609756 Cs

2. Chen K, Wang J, Pang J, et al (2019) MMDetection: Open MMLab Detection Toolbox and Benchmark. ArXiv190607155 Cs Eess
