# Supplementary material for: Pediatric age estimation from radiographs of the knee using deep learning
Source: Eur Radiol. 2022 Mar 1;32(7):4813–22. doi: 10.1007/s00330-022-08582-0 (PMC9213267; doi:10.1007/s00330-022-08582-0)
Supplement: Supplementary file 2 — (DOCX 51 kb) [file 330_2022_8582_MOESM2_ESM.docx]

**Annex 2**

***Neural Network for Age Estimation***

The ResNet-34 was used as backbone and feature extraction. As it is well known that the gender has a large impact on the maturity of the bones, the gender was used as additional input. Gender was first processed by two linear layers with a ReLU activation function and then added to the features of the ResNet-34. Then, another set of fully connected layers were used to regress the age (Figure A2).

The ResNet-34 was pretrained on the Image-Net dataset. As loss function, the L1 loss was used as this corresponds rather directly with the mean absolute error that is used as metric. Optimization was performed using the Adam optimizer. The batch size was set to 32. Early stopping was used on the cross-validation fold with a minimum delta of 0.01 and a patience of 25. To stabilize the training, the gradients of 5 batches were accumulated during training and Stochastic Weight Averaging was used (Annealing epochs of 10 with cosine annealing strategy).

As radiographs can be very large, all images were first resized to a size of 438 x 659. This rather odd number was used because the images were center cropped to a width of 384 (yielding an image size of 577 x 384) and maximally 1/8^th^ of the width of the original image should be cropped away.

During training several augmentations were used, which regularizes the network and helps its ability to generalize. The Albumentations framework was utilized for this. First, one of the following transformations were applied with 50% chance: Coarse Dropout (i.e. putting a black rectangle into the area) with maximum width and height of 1/10^th^ of the image, CLAHE with clipLimit of 4.0 and grid size of 8x8, random gamma transformation with limits between 70 and 130, random brightness contrast with brightness and contrast limits of 0.2, sharpening filter with alpha between 0.2 and 0.5 and lightness between 0.5 and 1.0 as well as a blur filter with blur limit of 7. After this, an elastic transform with no affine component and a rotation with maximum degree of 22 and with probability of 0.2 as well as a random flip was applied. The images were then intensity normalized to the range -1,1 by mean and standard deviation given by the ImageNet values.

During validation the images were resized to 438 x 659 and then center cropped to 577 x 384. Apart from an intensity normalization no other augmentations were used.

The network was developed using Python 3.7, PyTorch 1.4 and PyTorch Lightning 1.4 [1] and the OpenCV and Albumentation frameworks. The network was trained on commodity hardware (AMD Ryzen Threadripper 2950X with 128GB of RAM, a 1 TB M2.SSD drive and a NVidia TITAN RTX card, running Ubuntu 18.04 LTS). The source code of the experiments will be published in a repository on github [https://github.com/aydindemircioglu/knee.age].

1. Falcon et al. (2019) PyTorch Lightning


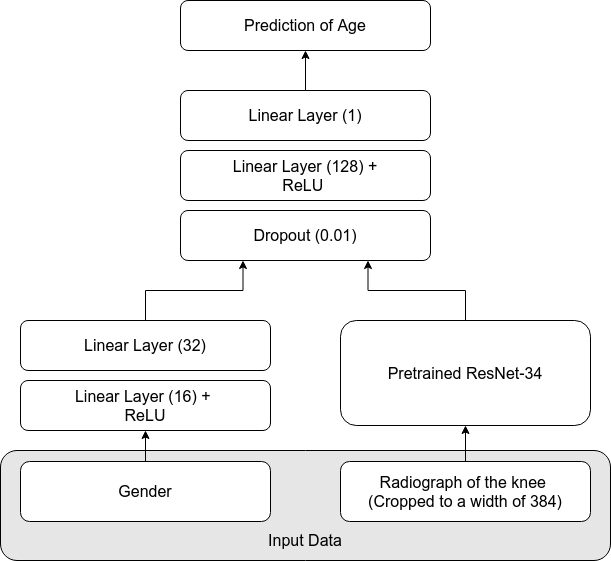


**Figure A2**: The network architecture for prediction of chronological age.
