# Supplementary material for: Pediatric age estimation from radiographs of the knee using deep learning
Source: Eur Radiol. 2022 Mar 1;32(7):4813–22. doi: 10.1007/s00330-022-08582-0 (PMC9213267; doi:10.1007/s00330-022-08582-0)

**Annex 3**

|  | Training cohort [N (F/M)] | Internal validation cohort [N (F/M)] | External validation cohort [N (F/M)] | All [N (F/M)] |
| --- | --- | --- | --- | --- |
| Age 0-1 | 14 (7/7) | 1 (0/1) | - | 15 (7/8) |
| Age 1-2 | 31 (20/11) | 4 (2/2) | 3 (1/2) | 38 (23/15) |
| Age 2-3 | 42 (16/26) | 5 (4/1) | 3 (2/1) | 50 (22/28) |
| Age 3-4 | 34 (16/18) | 8 (4/4) | 1 (0/1) | 43 (20/23) |
| Age 4-5 | 34 (13/21) | 3 (2/1) | 3 (1/2) | 40 (16/24) |
| Age 5-6 | 36 (10/26) | 9 (5/4) | 5 (2/3) | 50 (17/33) |
| Age 6-7 | 39 (16/23) | 6 (1/5) | 3 (1/2) | 48 (18/30) |
| Age 7-8 | 49 (17/32) | 5 (1/4) | 5 (1/4) | 59 (19/40) |
| Age 8-9 | 59 (26/33) | 6 (3/3) | 6 (2/4) | 71 (31/40) |
| Age 9-10 | 83 (39/44) | 13 (5/8) | 7 (3/4) | 103 (47/56) |
| Age 10-11 | 122 (57/65) | 16 (7/9) | 9 (7/2) | 147 (71/76) |
| Age 11-12 | 157 (75/82) | 13 (8/5) | 11 (3/8) | 181 (86/95) |
| Age 12-13 | 148 (68/80) | 18 (9/9) | 14 (6/8) | 180 (83/97) |
| Age 13-14 | 206 (92/114) | 34 (16/18) | 13 (6/7) | 253 (114/139) |
| Age 14-15 | 189 (73/116) | 34 (17/17) | 30 (9/21) | 253 (99/154) |
| Age 15-16 | 214 (100/114) | 27 (12/15) | 12 (5/7) | 253 (117/136) |
| Age 16-17 | 186 (94/92) | 23 (12/11) | 18 (6/12) | 227 (112/115) |
| Age 17-18 | 179 (85/94) | 27 (12/15) | 19 (10/9) | 225 (107/118) |
| Age 18-19 | 163 (65/98) | 24 (6/18) | 13 (6/7) | 200 (77/123) |
| Age 19-20 | 162 (76/86) | 28 (8/20) | 7 (4/3) | 197 (88/109) |
| Age 20-21 | 203 (100/103) | 23 (9/14) | 6 (4/2) | 232 (113/119) |

**Table A3**: Overview of all age classes in all three cohorts.

**Supplemental 4**

***Results of the cross-validation***

The maximum difference between true chronological and predicted ages was 5.4 years. The t-test indicated that the average of absolute differences between the predicted and the true ages are smaller than 1.0 years (p < 0.001). The Pearson correlation coefficient was high, with R = 0.96. The predictions were also compared to an ‘educated’ guess, which is the mean age of the cohort. This guess yielded a MAE of 3.48 ± 2.71 years during cross-validation and is thus clearly inferior to the network model. The maximum difference in age was 13.7 years, which corresponds to the difference of the youngest patient (0.27 years) and the mean of the training cohort.

**
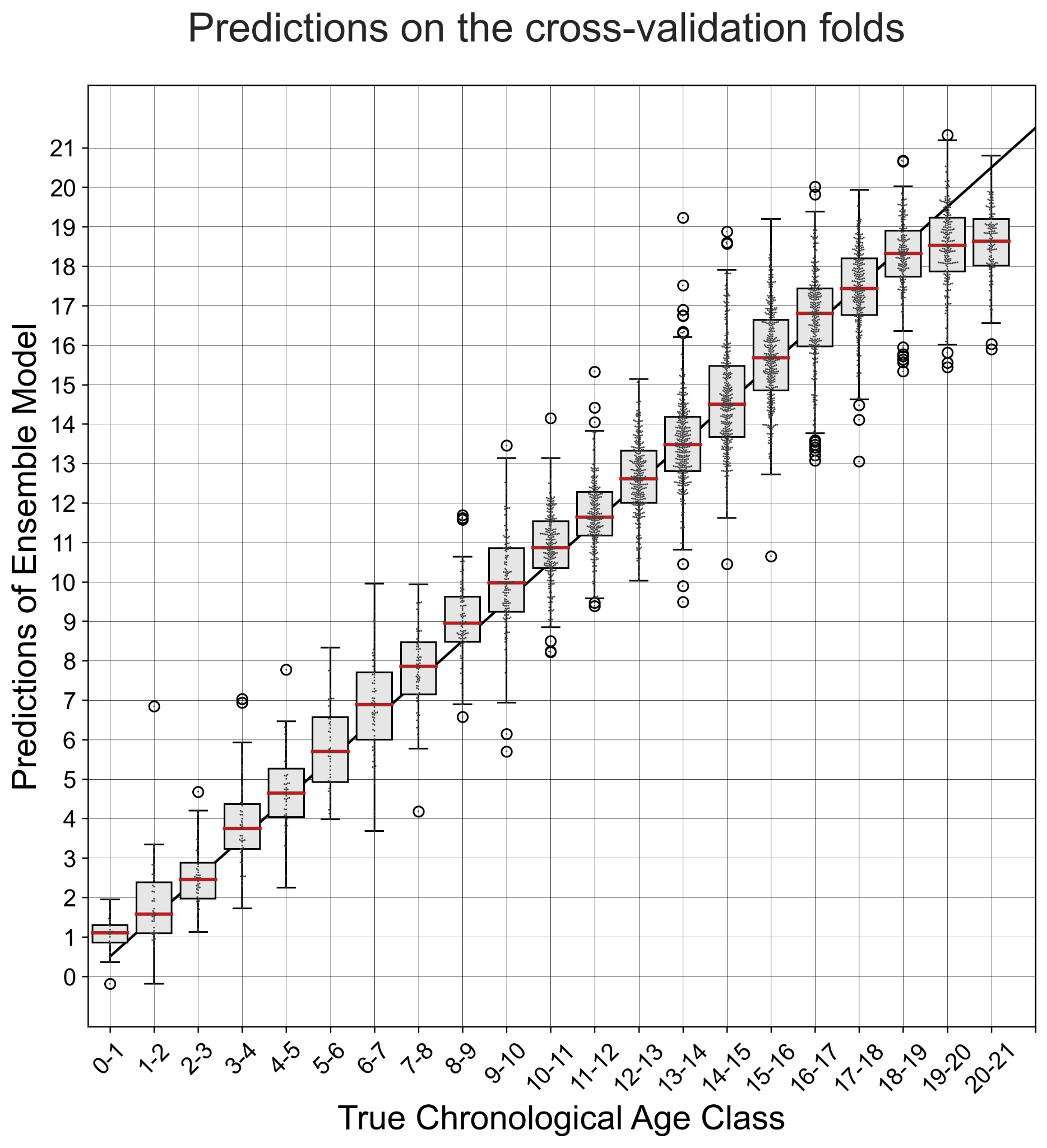
**


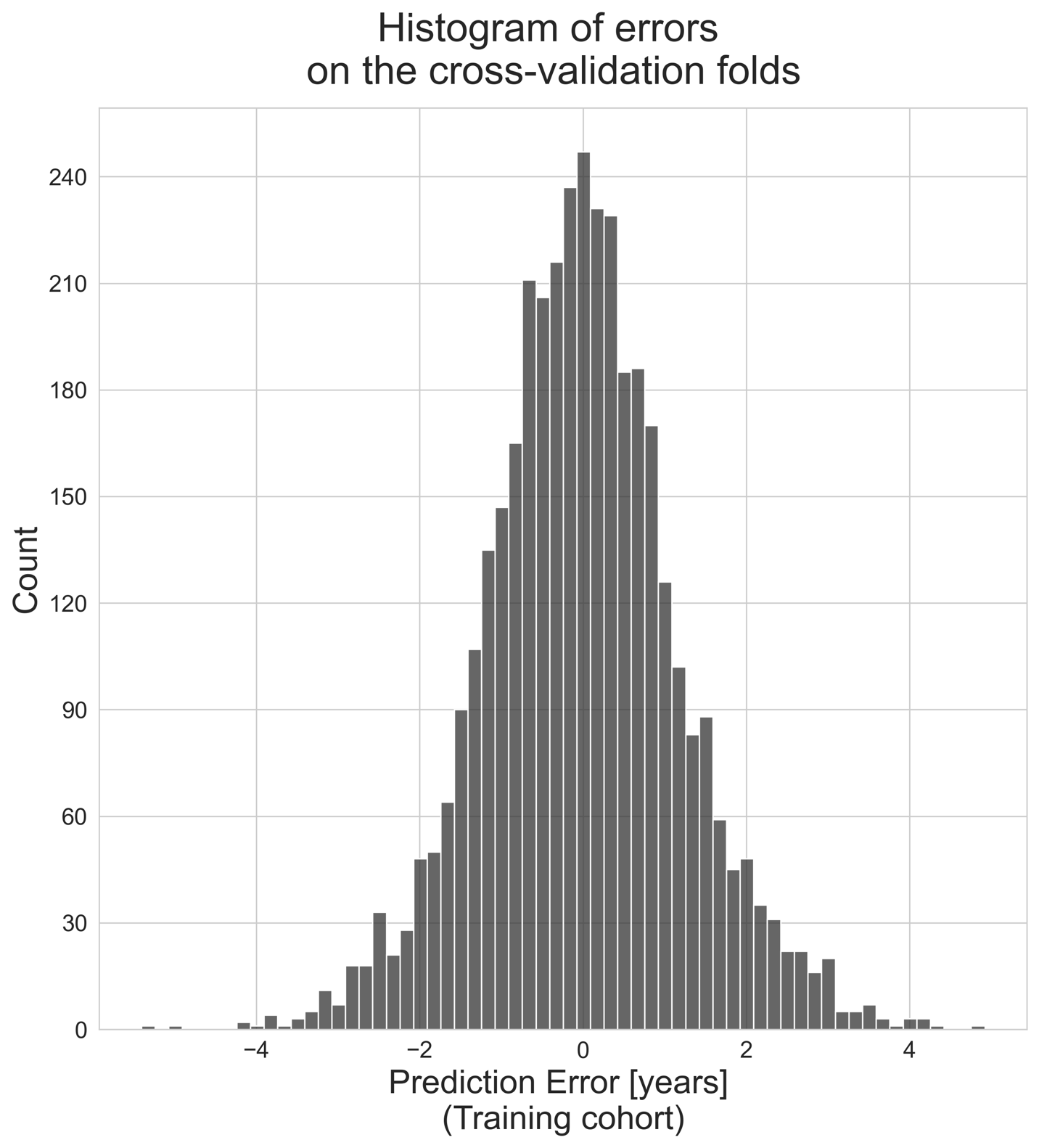

Supplement: Supplementary file 3 — (DOCX 706 kb) [file 330_2022_8582_MOESM3_ESM.docx]
