# Supplementary material for: Pediatric age estimation from radiographs of the knee using deep learning
Source: Eur Radiol. 2022 Mar 1;32(7):4813–22. doi: 10.1007/s00330-022-08582-0 (PMC9213267; doi:10.1007/s00330-022-08582-0)
Supplement: Supplementary file 4 — (DOCX 691 kb) [file 330_2022_8582_MOESM4_ESM.docx]

**Annex 4**

***Results of the cross-validation***

The maximum difference between true chronological and predicted ages was 5.4 years. The t-test indicated that the average of absolute differences between the predicted and the true ages are smaller than 1.0 years (p < 0.001). The Pearson correlation coefficient was high, with R = 0.96. The predictions were also compared to an ‘educated’ guess, which is the mean age of the cohort. This guess yielded a MAE of 3.48 ± 2.71 years during cross-validation and is thus clearly inferior to the network model. The maximum difference in age was 13.7 years, which corresponds to the difference of the youngest patient (0.27 years) and the mean of the training cohort.

**
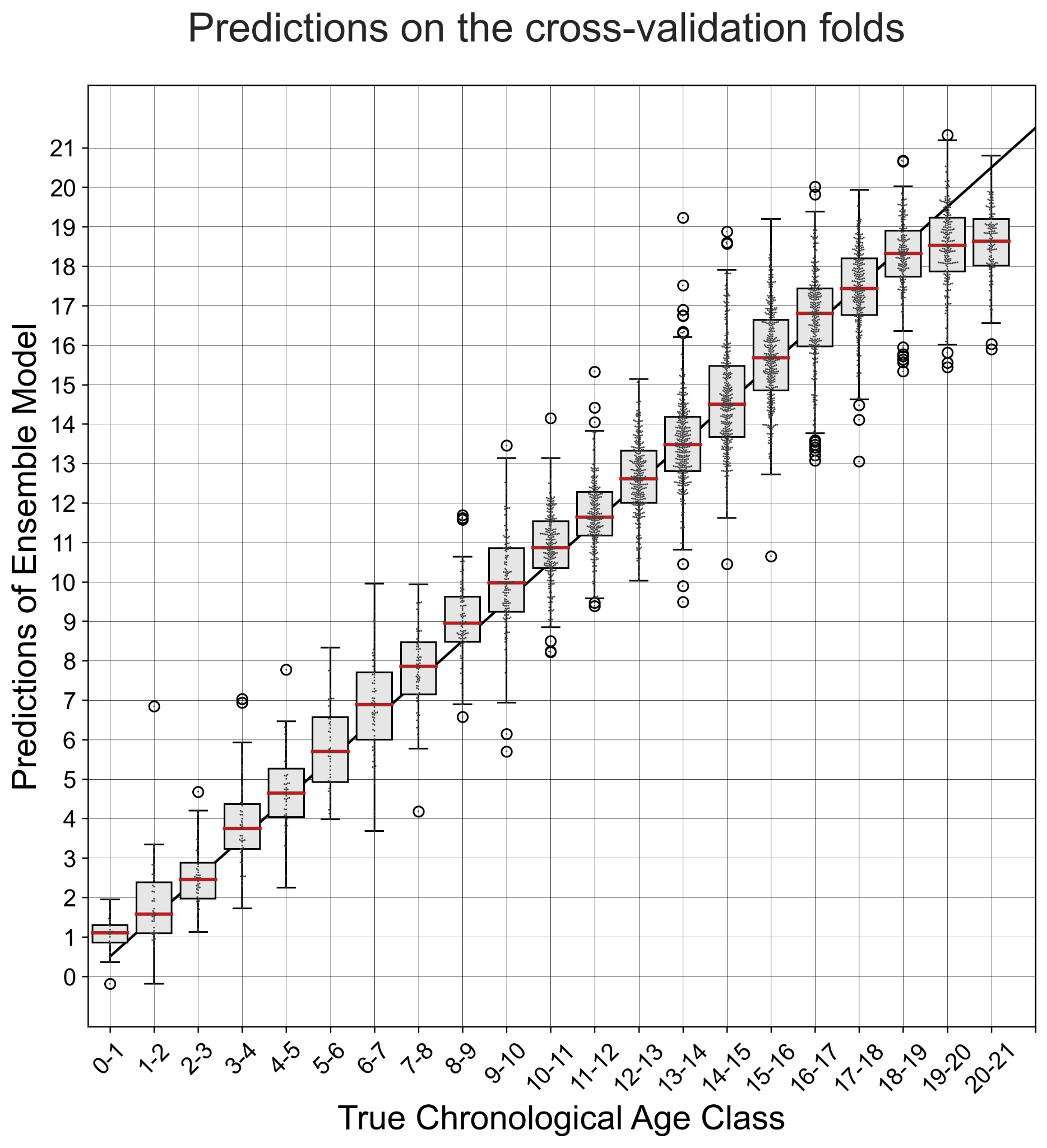
**

**Figure A4.1**: Box-plot of the predictions of the network evaluated on the cross-validation test folds. In the boxplots, for each true chronological age class, a corresponding box with whiskers for the corresponding network predictions was drawn. The median is marked by a red bar, while the whiskers extend to the points inside the 1.5*Interquartile range (IQR). In addition, all samples were marked by small dots. Outliers are marked with a circle.


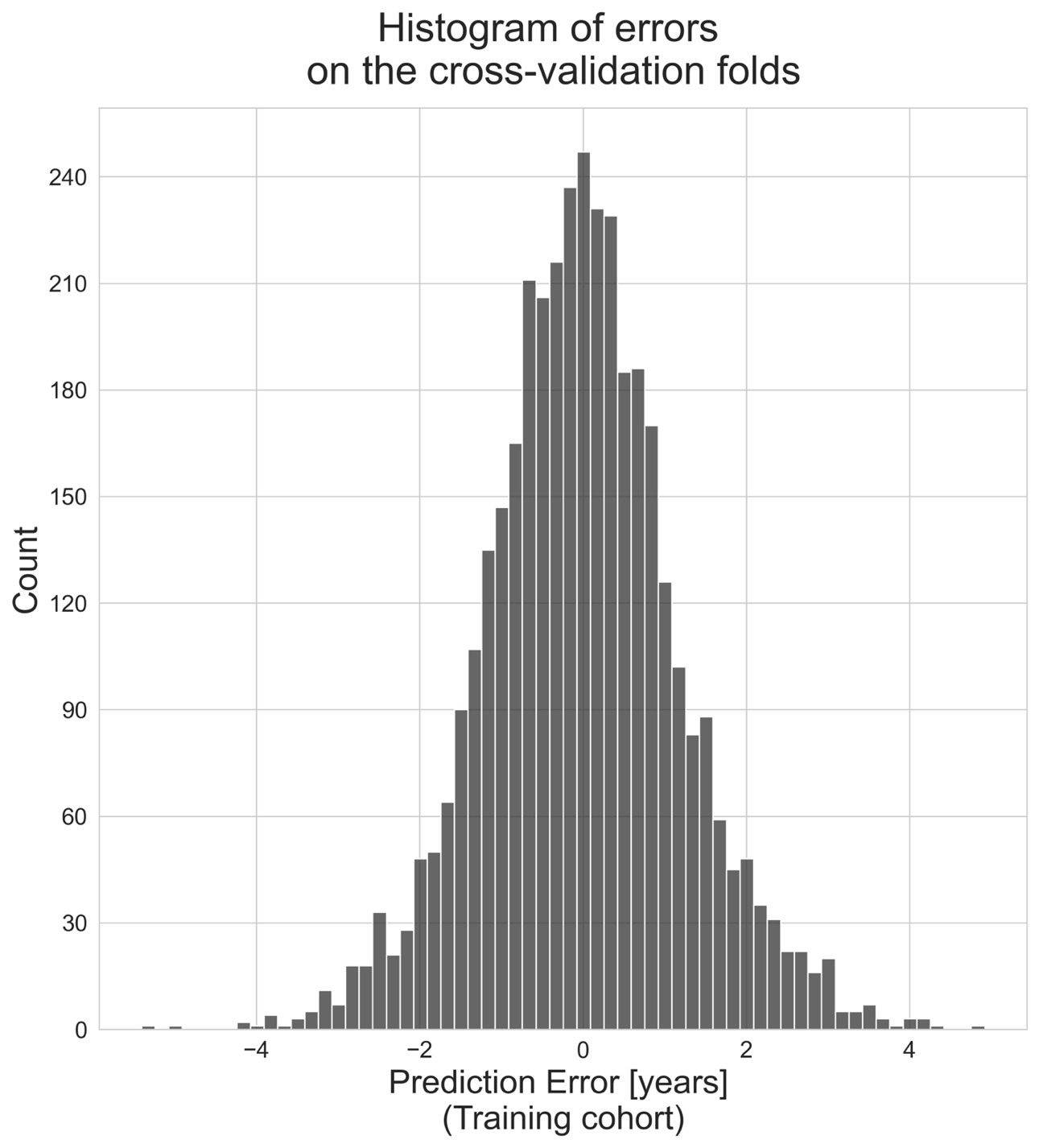


**Figure A4.2**: A histogram of the prediction errors on the cross-validation test folds.
